# Necrotizing soft tissue infection: clinical characteristics and outcomes at a reconstructive center in Jilin Province

**DOI:** 10.1186/s12879-017-2907-6

**Published:** 2017-12-28

**Authors:** Jing-Chun Zhao, Bo-Ru Zhang, Kai Shi, Xi Zhang, Chun-Hui Xie, Jian Wang, Qing-Hua Yu, Xin-Xin Gao, Lei Hong, Jia-Ao Yu

**Affiliations:** Burns and Plastic Reconstruction Unit, the First Hospital of Jilin University, No. 71 Xinmin Street, Changchun, 130021 China

**Keywords:** Necrotizing fasciitis, Surgical debridement, Treatment, Extremity

## Abstract

**Background:**

To investigate the clinical characteristics and treatment outcomes in necrotizing fasciitis (NF) patients in a reconstructive unit in northeastern China.

**Methods:**

Medical records of patients diagnosed with and treated for NF in the extremities from November 2013 to December 2016 were retrospectively reviewed. Demographic data, clinical presentation, duration of signs and symptoms, location of infection, predisposing factors, causative microbiological organisms, laboratory risk indicator for necrotizing fasciitis (LRINEC) score, number of surgical debridements, length of hospital stay, treatments, and outcomes were recorded.

**Results:**

A total of 39 consecutive patients were treated for severe NF (32 male and 7 female). Diabetes mellitus and blunt trauma were the most common risk factors (13 and 9 cases, respectively). The positive predictive value of the LRINEC score in NF diagnosis was 46.2%. Mean duration of signs and symptoms was 4.6 days. *Staphylococcus aureus* was the most commonly isolated bacteria (20 cases). All patients underwent their first debridement within 12 h of presentation (mean, 4.6 h). Mean number of surgical treatments was 2.8 (range, 2–5) per patient, including debridements. All patients survived, and mean length of hospital stay was 30.81 (range, 21–43) days. Three patients underwent limb amputation.

**Conclusions:**

In our clinical experience, early detection and aggressive debridement are the cornerstones of NF treatment. Antibiotic therapy and intensive care support is essential in severe cases of NF. Anaerobic tissue culture and frozen section biopsy could be adopted as routine tests for diagnosis and decision-making in NF. These findings should inform clinical decisions about the treatment of individual patients with NF.

## Background

Necrotizing fasciitis (NF) is a severe and potentially life-threatening soft tissue infection characterized by rapidly progressive necrosis of fascia and subcutaneous tissue along the fascial planes. NF is most commonly seen in lower extremities, followed by abdomen and perineum [1]. NF affects an estimated 0.4 to 1 person in 100,000 per year and accounts for substantial mortality and morbidity. Mortality associated with NF is reported at 11 to 36% [2]; among patients surviving NF, extensive scarring and physical and psychosocial impairment may lead to isolation and profound psychological problems [3].

Clinical presentation of NF varies from erythema, local warmth, swelling, and tenderness in the infected site in early stage to skin ischemia with blisters and hemorrhagic bullae and gas formation leading to crepitus in the overlying skin in advanced infection [4]. In the fulminant form of NF, patients deteriorate within a few hours, showing extensive necrosis of soft tissue, signs and symptoms of severe septic shock, and multiple organ dysfunction syndrome [2].

Several risk factors with prognostic significance have been identified in NF, including demographic factors (advanced age), trauma, chronic disease (diabetes mellitus, peripheral vascular disease, hypotension, immunodeficiency, chronic renal failure, liver cirrhosis), and lifestyle choices (intravenous drug abuse, alcoholism, obesity) [5–11].

A patient’s clinical characteristics, surgical exploration, and microbiological and histopathological analysis of soft tissue are the gold standard for diagnosis of NF. In equivocal cases, imaging (plain radiography, computer tomography [CT] scan, ultrasonography, magnetic resonance imaging [MRI]) is important for disease diagnosis and assessment of infection severity [12].

Treatment of NF includes intravenous administration of empirical broad-spectrum antibiotics and emergency surgical debridement. Importantly, ischemia and hypoxia may compromise delivery of antibiotics to the infection site; therefore, conservative treatment with antibiotics alone is not recommended [4]. The goal of surgical intervention is to remove all necrotic tissues, including muscle, fascia, and skin, to preserve viable skin, and achieve hemostasis. Postoperative wound management and proper nutritional support are vital for a patient’s survival.

The symptoms of NF disease are not consistent and misdiagnosis as cellulitis or abscess is common. As NF is a devastating disease, healthcare providers require best evidence from clinical care when making decisions about the treatment of individual patient. Therefore, to inform clinical practice, we describe the clinical characteristics, diagnosis, and treatment of NF in a convenience case series of patients in Jilin Province, which lies in the mainland of northeastern China.

## Methods

### Study population

Patients diagnosed with and treated for NF at the First Hospital of Jilin University from November 2013 to December 2016 were eligible for this study. Inclusion criteria were 1) primary diagnosis of NF, and 2) infection in the extremities. Patients with other infectious disease, such as cellulitis, or missing information were excluded.

Medical records of discharged or deceased patients with a primary diagnosis of NF were identified by a computerized search and retrospectively reviewed. Data including age, sex, body mass index (BMI), clinical presentation, duration of signs and symptoms, location of infection, predisposing factors, causative microbiological organisms, on-admission laboratory results, number of surgical interventions, length of hospital stay, treatments, and final outcomes, were collected. The use of the Laboratory Risk Indicator for Necrotizing Fasciitis (LRINEC) score as an ancillary method in the diagnosis of NF was also investigated. A LRINEC score of 6 or higher was considered predictive of the presence of NF.

### Management of NF

NF was initially diagnosed by two equally experienced senior surgeons independently based on intra-operative findings in accordance with previous reports [4, 11, 13]. Disagreements were resolved with the help of a third professor. Criteria included 1) presence of grayish necrotic fascia or lack of facial bleeding; 2) lack of tissue resistance of normally adherent fascia to blunt finger dissection, and 3) purulent discharge resembling foul-smelling dishwater.

At presentation, blood cultures and liver function tests were performed and arterial blood-gas analysis, baseline full blood count, and urea, electrolyte, and C-reactive protein levels were measured. On admission, the pus or wound secretion with skin and soft tissue infection was also collected, bacteria cultures and pathogen identification as well as drug sensitivity test were adequately performed. A combination antibiotic regimen consisting of intravenous meropenem (0.5 g, three times a day, Sumitomo Dainippon Pharma Co., Ltd., Osaka, Japan) and vancomycin (0.5 g, four times a day, Eli Lilly Japan K.K, Seishin Laboratories, Japan) was initiated. Antibiotic regimens were adjusted according to the positive results of blood and/or wound swab cultures. Supportive therapies, including maintenance of fluid and electrolyte balance, blood product transfusion, blood sugar control, and nutritional support were provided as needed. In some cases, resuscitation with intravenous fluids and colloids was necessary.

Emergency surgical debridement was performed in all patients. Skin incision was performed in the longitudinal direction along the muscle-fascial layers of the affected area, until healthy fascia was encountered. Complete surgical excision of all infected and necrotic tissue including a 5–10 mm margin of healthy fascia was performed.

In the immediate post-operative period, patients were transferred to the intensive care unit (ICU) for close monitoring of disease progression until they were haemodynamically stable.

Post-operatively, serial dressing changes were carried out by experienced attending surgeons, with evaluation of surgical wounds and tissue viability. Hand-made gauze-based negative pressure wound therapy (NPWT) was applied. Definitive closure was performed when the wound was free of recurrent or ongoing infection.

## Results

This case series included 39 consecutive patients treated for severe NF; 33 patients had NF of the lower limbs, and 6 patients had NF of the upper limbs. Thirty-seven patients were adults (mean age: 45.6 years), and two patients were children (aged 7 months and 30 months). The male-to-female ratio was 4.6:1 (32 male and seven female). Twenty-seven patients were farmers, five patients resided in the city, three patients were unemployed, one patient was retired, one patient was a student, one patient was a 7-month old infant, and one patient was a 30-month old child. Most patients were *Han* nationality (35 patients), three patients were *Manchu*, and one patient was *Mongol*. Mean BMI of the adult patients was 22.9 kg/m^2^. In this case series, the positive predictive value of the LRINEC score in diagnosis of NF was 46.2% (Table 1).

**Table 1:** Patients’ demographic and clinical characteristics (*N* = 39)

Variable	n (%)
Age (years)	45.6
Sex	
Male	32 (82.05%)
Female	7 (17.95%)
Duration of symptoms (days)	4.6
Affected sites	
Upper extremities	6 (18.18%)
Lower extremities	33 (84.62%)
Causative organism(s)	
Gram positive bacteria	
*Staphylococcus aureus*	18 (46.15%)
*Methicillin-resistant Staphylococcus aureus*	2 (5.13%)
*Enterococcus faecalis*	1 (2.56%)
*Enterococcus faecium*	2 (5.13%)
*Streptococcus anginosus*	3 (7.69%)
*Staphylococcus epidermidis*	4 (10.26%)
*Streptococcus agalactiae*	1 (2.56%)
*Coagulase-negative staphylococci*	1 (2.56%)
*S. constella subsp. constellatus*	1 (2.56%)
*Enterococcus avium*	1 (2.56%)
Gram negative bacteria	
*Klebsiella pneumoniae*	2 (5.13%)
*Enterobacter cloacae*	5 (12.82%)
*Pseudomonas aeruginosa*	3 (7.69%)
*Acinetobacter baumannii*	4 (10.26%)
*Escherichia coli*	5 (12.82%)
Negative results of culture	6 (15.38%)
Comorbidities	
Diabetes mellitus	13 (33.33%)
Liver cirrhosis/Chronic hepatitis	1 (2.56%)
Burns	2 (5.13%)
Insect bite	1 (2.56%)
Rash	1 (2.56%)
Blunt trauma	9 (23.08%)
Cancer	1 (2.56%)
Pressure injury	2 (5.13%)
Erythema nodosum	1 (2.56%)
Skin infection/adscess	5 (12.82%)
Use of nonsteroidal anti-inflammatory drugs	1 (2.56%)
Idiopathic/no comorbidities	5 (12.82%)
LRINEC score positive	18 (46.15%)

*LRINEC* laboratory risk indicator for necrotizing fasciitis

Among the 39 patients, 34 patients had risk factors for NF. Diabetes mellitus was the most common risk factor (13 patients), followed by blunt trauma (9 patients), skin infection or abscess (five patients), burns (two patients), pressure injury (two patients), rash (one patient), cancer (one patient), liver cirrhosis (one patient), erythema nodosum (one patient), use of nonsteroidal anti-inflammatory drugs (one patient), and insect bite (one patient); trauma with diabetes mellitus was observed in three patients.

Clinical characteristics on presentation included spreading erythema, swelling, and pain. Duration of signs and symptoms varied from 1 to 8 days (mean 4.6 days). Twenty-one patients allowed their condition to deteriorate for 3–4 days after the onset of NF before seeking treatment (Table 2). Six patients underwent inadequate debridement at local hospitals. Three patients suffered from septic shock on admission, cardiac dysfunction was found in one patient related to NF (New York Heart Association class II).

**Table 2:** Clinical presentation (*N* = 39)

Symptoms or signs	n (%)
Local symptoms	
Erythema	30 (76.92%)
Swelling	35 (89.74%)
Pain or tenderness	37 (94.87%)
Blisters	22 (56.41%)
Hemorrhagic bullae	20 (51.28%)
Crepitus	11 (28.21%)
Skin necrosis	36 (92.31%)
Systemic symptoms	
Fever (> 38.0 °C)	14 (35.90%)
Tachycardia (> 100 beats/min)	19 (48.72%)
Hypotension (SAP < 100 mmHg)	16 (41.03%)
Tachypnea (> 20/min)	18 (46.15%)

*Staphylococcus aureus* was the most frequently isolated pathogen, occurring in wound swab cultures from 20 patients. Antibiotic resistance in isolated pathogens were found in three patients, including two patients with methicillin-resistant *Staphylococcus aureus* (MRSA) and resistance to cephalosporin was noted in one patient. Polymicrobial, gram-positive monomicrobial, and gram-negative monomicrobial organisms were found in 19, 11 and 3 patients, respectively. Wound swab cultures were negative in 6 patients. No marine-related organisms or fungus was noted.

All suspected NF patients underwent surgical debridement within 12 h of presentation (mean, 4.6 h). All the patients were transferred to the intensive care unit (ICU) for post-operative intensive care. The mean length of stay in the ICU was 9.8 days (range, 7–13). Four patients returned to the operating room for reexploration.

Nineteen patients received hand-made gauze-based NPWT following debridement. Definitive soft tissue reconstructive procedures, either with primarily suture (three patients) or extensive meshed split-thickness skin grafting (30 patients), were performed when the infected necrotic tissue was stabilized. Three patients underwent limb amputation. A mean of 2.8 (2–5) surgical treatments was performed per patient.

**Fig. 1:**
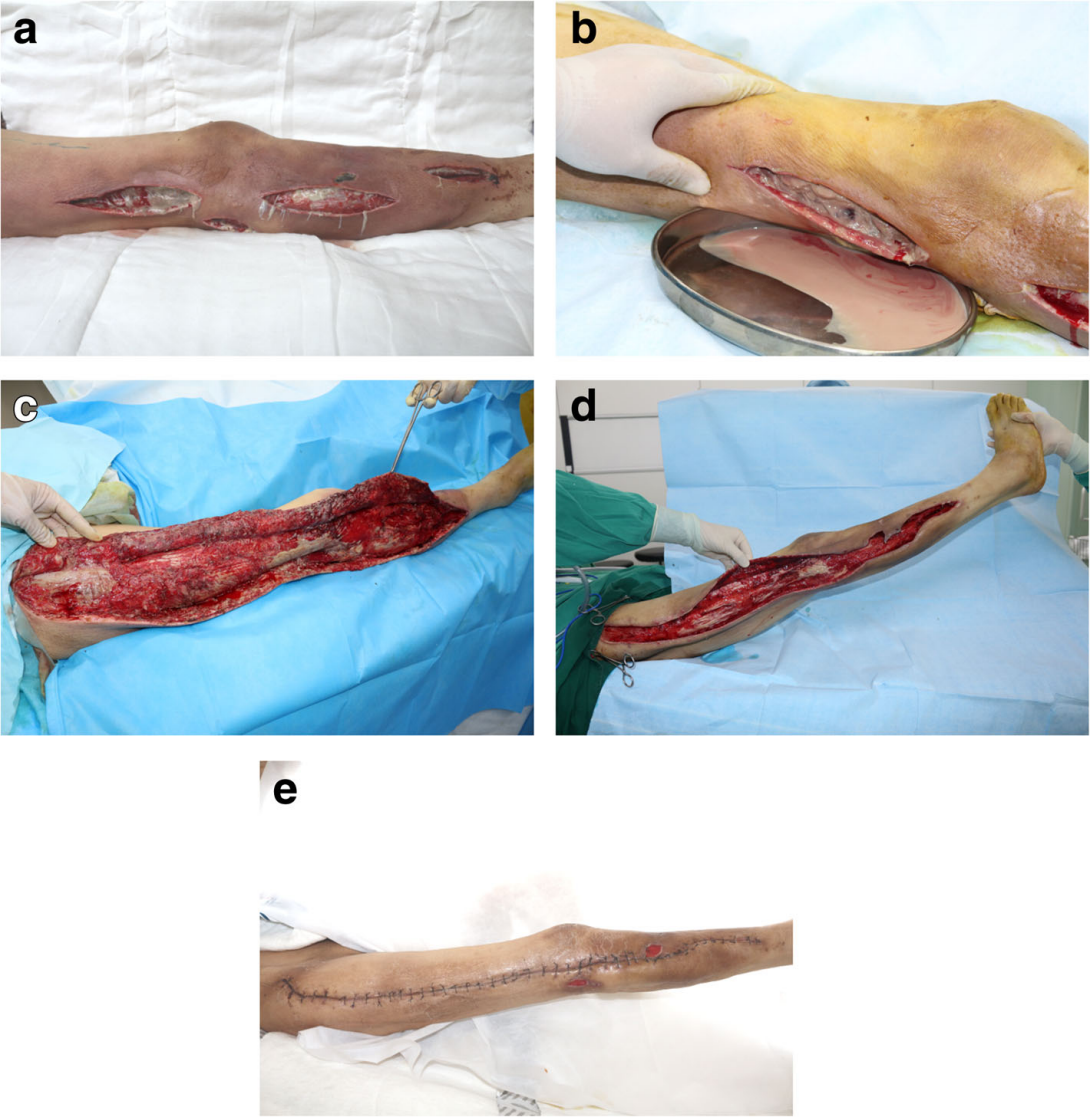
A 57-year-old diabetic male experienced trauma to the right lower limb that resulted in immediate pain, swelling, and subcutaneous congestion. The patient did not seek treatment; therefore, the swelling and pain became widespread. The patient attended a local hospital where he underwent multifocal debridement and received antibiotic therapy, but his condition did not respond. Ten days later, the patient was transferred to our department for further definitive treatment. **a** the right lower limb was painful, with swelling and erythema. Multifocal debridement was performed, and necrotic tissue was exposed. **b** Foul smelling dishwater pus was present. **c** Aggressive debridement was performed revealing extensive necrosis of fascia and subcutaneous tissue. **d** Following surgery, the patient underwent negative pressure wound therapy and debridement at the bedside. **e** The clean wound bed was closed using sutures; the patient was discharged when all the sutures were removed, 2 weeks postoperatively

**Fig. 2:**
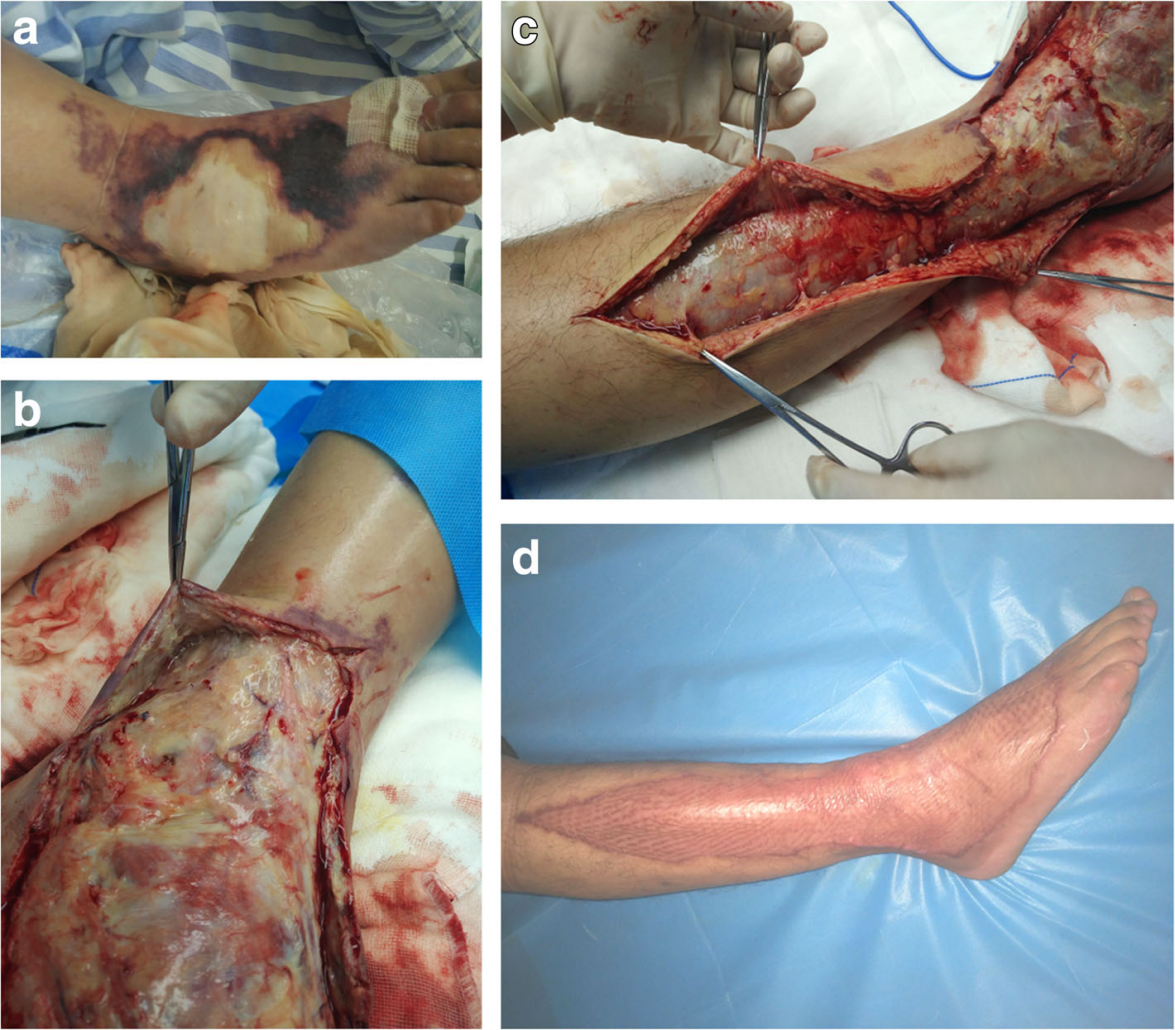
A 37-year-old male suffering from aggressive pain and erythema of the right lower leg and foot for no identifiable reason was referred to our department for further definitive diagnosis and treatment after 1 day. **a** Swelling, subcutaneous congestion, necrosis and tenderness were found on the right foot and lower leg, with hypotension (85/53 mmHg), tachypnea (20 beats per minute), and tachycardia (113 beats per minute). **b**-**c** Emergency debridement was performed immediately after admission, and widespread necrosis was found. Debridement was repeated on day 4 and 9 after admission. **d** Skin graft was used to close the wound 1 month after the initial debridement

**Fig. 3:**
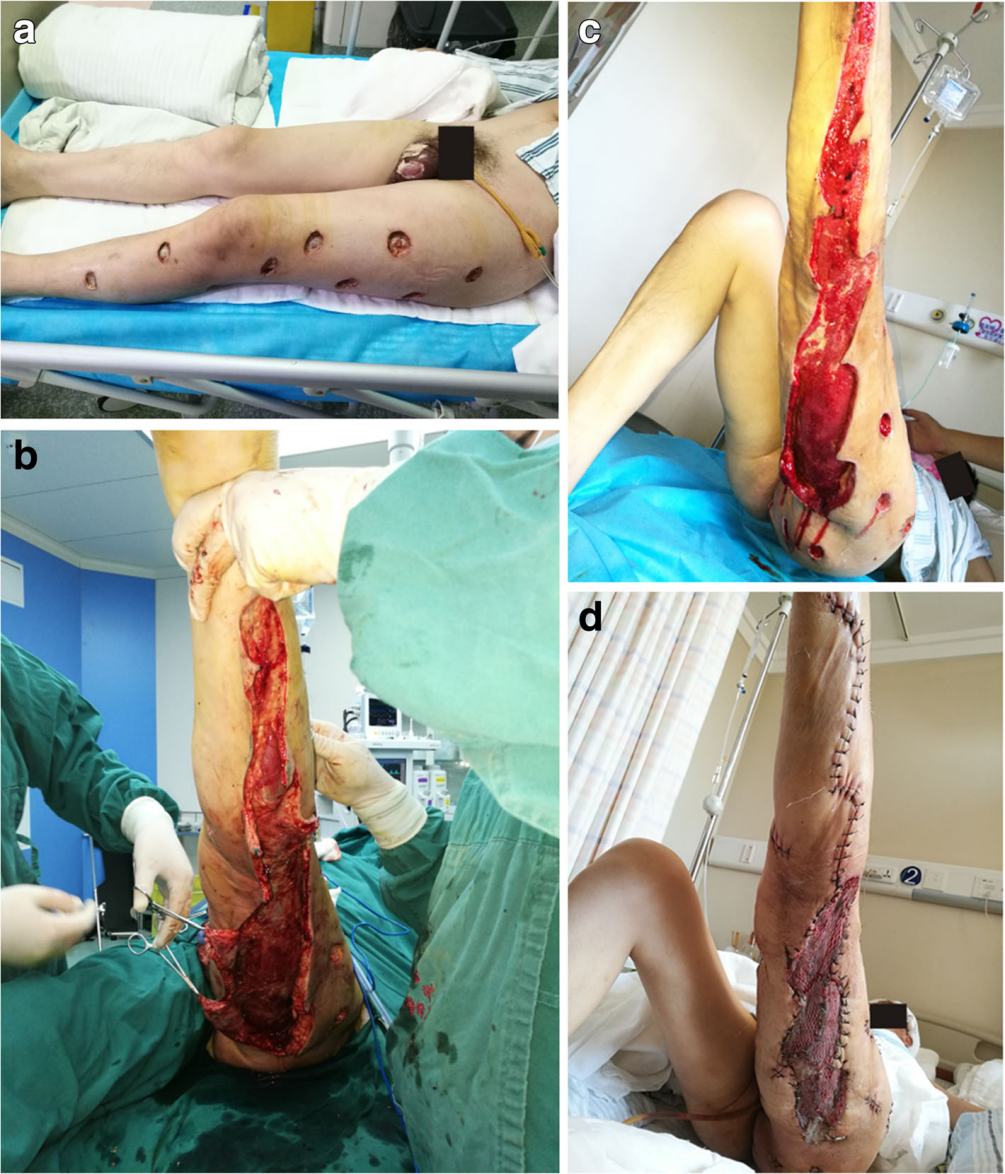
A 48-year-old male suffering from pain, swelling, and subcutaneous congestion to the left lower limb and scrotum received antibiotic therapy and multifocal debridement at a local hospital, but his condition did not respond. Ten days later, and after two to four episodes of fever (39–40 °C), the patient was transferred to our department for further definitive treatment. **a** Multifocal debridement was performed on the left lower limb, and necrotic tissue was exposed. **b** Aggressive debridement was performed revealing extensive necrosis of fascia and subcutaneous tissue. **c** The patient underwent negative pressure wound therapy and debridement at the bedside. **d** The clean wound bed was closed using sutures and skin grafting

All the patients survived and were discharged after treatment; mean length of hospital stay was 30.81 (range, 21–43) days. Three representative clinical cases will be presented (Figs. 1, 2 and 3).

## Discussion

This case series described the characteristics of NF patients in Jilin Province, China. Findings showed that most NF patients were male farm laborers that did not seek immediate treatment. This suggests that a wider range of medical insurance coverage is necessary to enable rural patients to seek timely treatment. Those that did attend local hospitals received inadequate debridement and treatment. In accordance with previous study [14], diabetes mellitus was the most frequent comorbidity in this case series, followed by a history of blunt trauma.

Although group A β-haemolytic streptococci (*Streptococcus pyogenes*) is classically associated with NF, *Staphylococcus aureus* was the most frequently isolated pathogen from wound secretion cultures in this study (20/39). *Staphylococcus aureus* is a remarkable pathogen with emerging virulence. It has been an uncommon cause of NF, however, the incidence with *Staphylococcus aureus* identified as the primary pathogen of monomicrobial NF is on the rise, and community-associated MRSA has emerged as the predominant causative agent in recent years [15–17]. Recent study shows that *spa* type t008 may be responsible for the increasing incidence of *Staphylococcus aureus* NF infections [18].

Jilin Province is an agricultural area, accounting for the high prevalence of males and farm laborers in our patient population. In Asia, NF is frequently caused by seafood ingestion or exposure or injury in a marine environment [8], and pathogenic bacteria include *Vibrio* spp., *Aeromonas* spp., and *Shewanella* spp. [19]. In the present case series, there were no bacterial infections from aquatic species, likely due to the lifestyle and occupation of the patients; the majority of patients were farmers (27/39) or urban workers (5/39).

NF is a lethal infection characterized by an insidious clinical presentation and progressive tissue destruction. According to the clinical manifestations of NF, Wang et al. [20] proposed a staging system based on disease progression: early (stage 1) characterized by tenderness to palpitation, erythema and swollen, warm skin; intermediate (stage 2), characterized by blisters, bullae, skin fluctuance and induration; and late (stage 3), characterized by hemorrhagic bullae, crepitus, and skin necrosis. The majority of patients (26/39) included in the present case series were classified as stage 3.

Morgan [5] classified NF into four types based on causative microbiological organisms; type I caused by polymicrobial/synergistic pathogens, type II caused by monomicrobial and gram-positive pathogens, type III caused by gram-negative monomicrobial, bacterial infections from aquatic species, and type IV caused by fungal pathogens; they identified type I in 70% of cases. In contrast, in the present case series, type I was identified in 57.58% of cases, monomicrobial infection was identified in 42.42% (33.33 and 9.09% for gram-positive and gram-negative bacteria, respectively) of cases. No type III and type IV NF was noted.

Primary management of NF involves emergency aggressive surgical debridement of affected tissues; in fulminant forms of NF, inadequate or delayed surgery can be fatal [21]. Surgical debridement should be repeated over the following 24 h, depending on the clinical course of the infection and the patients’ vital signs [22]. In the present case series, mean duration of signs and symptoms before seeking treatment was 4.6 days; therefore, infection had spread and the disease had progressed in many patients. All patients were treated successfully at our hospital and discharged; only four patients required a second-look operation, probably because initial debridement was early and aggressive.

Limb amputation was performed in three patients. Two cases were due to delayed presentation (11 and 14 days after initial onset), whereby necrosis had become severe and widespread, involving the soft tissue and underlying muscles and progressing to septic shock. In the other patient, amputation was required due to ulceration of squamous cell carcinoma. These findings emphasize that early detection and prompt appropriate management is essential to prevent disease progression, minimize the need for repeat surgeries, and reduce the amputation rate in NF.

NPWT is applied in the management of NF following debridement as it provides combined benefits of continuous wound cleansing and promotes the formation of granulation tissue. In our case series, hand-made gauze-based NPWT was used to reduce the economic burden on the patients.

CT, ultrasound, and MRI are useful tools for diagnosis of NF, but they are time-consuming and expensive and may not be readily available in an emergency setting. In our experience, these imaging modalities are useful in haemodynamically stable patients. Laboratory data can also provide useful supplemental information and raise the suspicion for NF when the diagnosis is uncertain. Wong et al. [23] developed the LRINEC score to identify patients at higher risk for NF from non-necrotizing soft tissue infections. The LRINEC score is based on six basic laboratory parameters at the time of presentation: white blood cell count, hemoglobin, C-reactive protein, serum sodium, serum creatinine, and serum glucose. The LRINEC score has been verified as a useful tool for detecting NF as well as predicting mortality of NF [24, 25], while other studies show that the LRINEC scoring system is not applicable when treating NF and question its value as a diagnostic and prognostic tool [26, 27]. In the present study, we use LRINEC score as an ancillary method in the differentiating NF and non-NF related skin and soft tissue infection. The validation of LRINEC in our group of patients showed a reduced sensitivity of 46.2%, which was comparable with previous research [28].

Hyperbaric oxygen therapy and intravenous immunoglobulin administration have shown benefits in the management of NF [29, 30]. However, these treatment modalities have not been adopted in our clinical setting as high quality clinical studies are limited and there is currently insufficient evidence to recommend the use of these treatments in patients with NF [12, 31].

Our study was associated with several limitations. First, it was a single center study with a small sample size of patients with NF in the extremities; NF that involved the head, neck, and perineum was excluded. Second, anaerobic culture and histological and radiological results were not obtained to confirm the diagnosis of NF. Third, although repeated wound swab culture was performed, 15.38% results were negative. This may be because only aerobic culture was performed. The use of anaerobic culture and frozen section biopsy will be considered in future work.

## Conclusions

In our clinical experience, early detection and aggressive debridement are the cornerstones of NF treatment. Antibiotic therapy and intensive care support is also essential in severe cases of NF. Anaerobic tissue culture and frozen section biopsy could be adopted as routine tests for diagnosis and decision-making in NF. These findings should inform clinical decisions about the treatment of individual patients with NF.
